# Development and validation of a type 1 diabetes self-management scale for children

**DOI:** 10.1590/1806-9282.20252204

**Published:** 2026-07-31

**Authors:** Merve Aşkın Ceran, Murat Bektas, Beray Selver Eklioğlu

**Affiliations:** 1 Dokuz Eylul University, Health Science Institute, Department of Pediatric Nursing – İzmir, Türkiye.; 2 KTO Karatay University, Vocational School of Health Services – Konya, Türkiye.; 3 Dokuz Eylul University, Faculty of Nursing, Department of Pediatric Nursing – İzmir, Türkiye.; 4 Necmettin Erbakan University, Faculty of Medicine, Division of Pediatric Endocrinology – Konya, Türkiye.

**Keywords:** Children, Type 1 diabetes mellitus, Self-management, Psychometric, Development, Scale

## Abstract

**OBJECTIVE:**

The aim of this study was to develop a type 1 diabetes mellitus self-management scale for children and to assess its psychometric properties.

**METHODS:**

A methodological, descriptive, correlational, and cross-sectional study was conducted. The research sample comprised 212 children with diabetes aged 7–12 years. Content validity was evaluated by seven experts in pediatric endocrinology, pediatric nursing, public health nursing, and Turkish language. Descriptive statistics, correlation analyses, Cronbach's alpha, McDonald's omega, split-half reliability, and factor analyses were used.

**RESULTS:**

The Cronbach's alpha for the overall scale was 0.893, and for the subscales, it ranged from 0.757 to 0.845. The item-total score correlations ranged from 0.408 to 0.660. Exploratory factor analysis revealed a four-factor structure that explained 57.730% of the total variance, with item loadings ranging from 0.380 to 0.863. Confirmatory factor analysis supported the four-factor structure, with factor loadings ranging from 0.52 to 0.87 and acceptable fit indices: χ^2^/df=1.461, goodness-of-fit index=0.901, comparative fit index=0.939, incremental fit index=0.941, Tucker–Lewis index=0.926, and root mean square error of approximation=0.066.

**CONCLUSION:**

The validity and reliability analyses revealed that the scale is a valid and reliable measure in Turkish culture.

## INTRODUCTION

Diabetes mellitus (DM) is a chronic metabolic disorder caused by insufficient insulin secretion or reduced cellular insulin sensitivity, leading to impaired carbohydrate, fat, and protein metabolism^1,2^. The most common type in childhood is Type 1 diabetes mellitus (T1DM), which results from β-cell destruction and insufficient or absent insulin production, causing hyperglycemia^3,4^. According to the Type 1 Diabetes Index, T1DM is rapidly increasing worldwide. In 2020, an estimated 8.8 million people were living with T1DM, a number expected to reach 17.4 million by 2040. Globally, 1.52 million individuals under age 20 have T1DM, including about 29,000 children in Turkey^5^. Although T1DM can occur at any age, it is most common between ages 4–6 and 10–14, when autoimmune mechanisms and environmental factors intensify β-cell damage^6^. Contributing factors include delayed physical growth, advanced cognitive development, increased exposure to infections upon school entry, and hormonal and psychological changes during adolescence. A T1DM diagnosis causes lasting physical, emotional, and social changes in children, making self-management difficult^6^. Consequently, providing effective self-management education and ensuring lifelong support for children and their families are essential, as poor quality of life in individuals with diabetes is associated with reduced self-management, inadequate glycemic control, and increased complications^6-8^. Key self-management components include regular exercise, appropriate nutrition, medication adherence, avoidance of risky behaviors, prevention of complications, and glucose monitoring^9^. Effective self-management is essential for optimal glycemic and metabolic control, improved treatment adherence and self-care, reduced complications, and decreased healthcare costs^10^. Assessing the achievement of treatment goals is a key outcome of self-management follow-ups in T1DM^11^. Although several diabetes self-management studies and scales exist in Turkey, most focus on single dimensions or older age groups, and comprehensive tools for children are limited^12-15^. Therefore, a child-centered and holistic type 1 diabetes self-management scale for children (T1DMS-C) was developed for children ages 7–12, and this study aims to evaluate its psychometric properties.

## METHODS

### Ethical approval

This study was conducted in accordance with the principles set out in the Declaration of Helsinki. Ethics committee approval was obtained from the Pharmaceuticals and Non-Medical Device Research Ethics Committee (Decision number 2021/013). Written approval was obtained from the chief physician of the hospital where the study was conducted, with the number E-14567952-900-147026. Written and verbal consent was also obtained from the children and their parents.

### Purpose

This methodological, descriptive, comparative, and cross-sectional study was conducted to develop the T1DMS-C and evaluate its psychometric properties. The scale development process was carried out in accordance with internationally recommended stages, including item pool creation, expert review for content validity, pilot testing, construct validity testing through exploratory and confirmatory factor analyses, and reliability assessment^16,17^.

### Sample

The study sample included 212 children aged 7–12 who visited the pediatric endocrinology outpatient clinic of a university hospital. A minimum sample size of 200 participants was targeted, based on methodological recommendations suggesting that samples of approximately 200 are generally adequate for factor-analytic procedures in scale development studies^16-18^. A sample of 200 children was planned. Ultimately, 212 children who agreed to participate were included in the study.

### Scale development

A literature review was conducted to develop the items for the scale. To identify diabetes self-management and related concepts, the literature was searched using keywords such as self-management, diabetes self-management, childhood diabetes management subscales, and risk factors affecting blood glucose and complication management. Relevant scales and guidelines from the World Health Organization, the Turkish Endocrinology and Metabolism Association, and the American Diabetes Association were also reviewed. Based on these sources, the researchers created a 47-item draft scale; following expert review, one item was removed, and the scale was finalized with 46 items.

### Content validity

To evaluate content validity, feedback was obtained from seven experts, including a pediatric endocrinologist, four pediatric diabetes nurses, a public health nurse, and a Turkish language specialist. Expert agreement was assessed using the Davis method to determine whether each item and the overall scale adequately reflected diabetes self-management in children.

**Figure 1:**
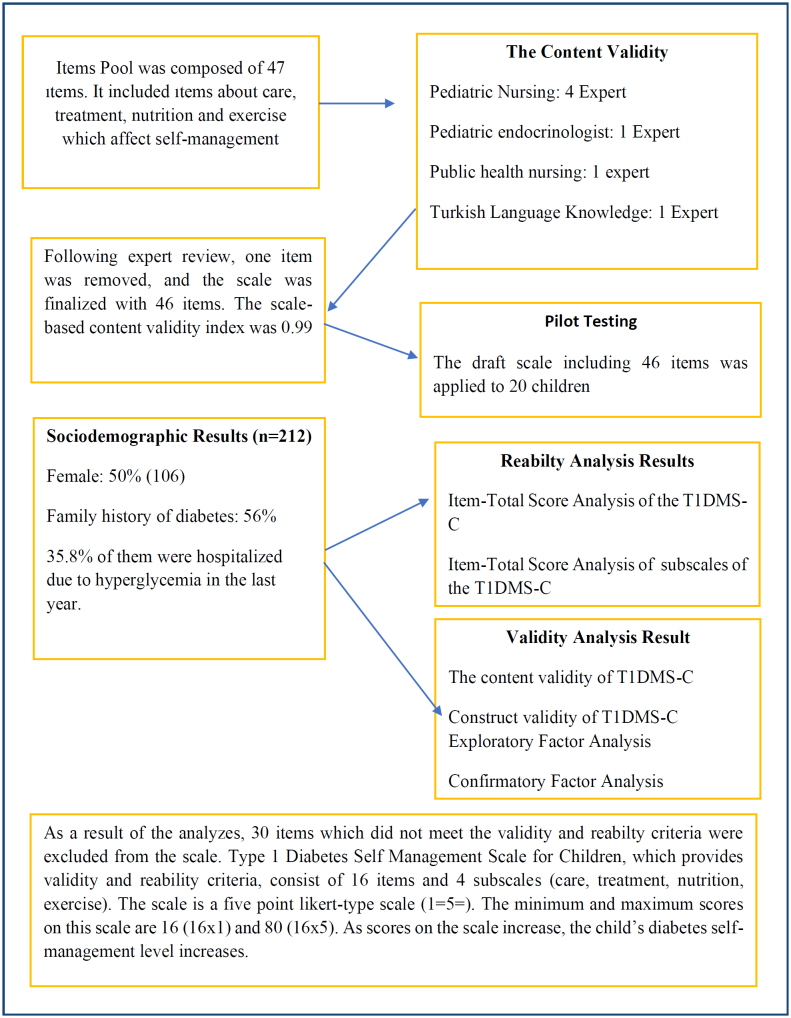
Development and validation of a type 1 diabetes self-management scale for children.

One item was removed from the scale, which originally contained 47 items, based on expert opinion, leaving 46 items. Content validity was examined at both the item level (I-CVI) and the scale level (S-CVI). According to the Davis method, an agreement level of at least 0.80 is required. One item did not meet this criterion and was removed from the scale. The remaining items showed I-CVI values ranging from 0.98 to 1.00, and the overall S-CVI value was 0.99. Therefore, the revised item set was considered appropriate for the pilot study. Expert evaluation is an essential step in scale development, as it ensures that items represent the intended construction and provide evidence of content validity^16,17^ (Figure 1).

### Pilot study

Following expert review, the draft scale was pilot tested with 20 children who were not included in the main study sample but had similar characteristics. This pilot application aimed to evaluate the clarity and comprehensibility of the items, the time required to complete the scale, and whether any items were interpreted differently by participants. No negative feedback was received, and the scale was completed in approximately 15–20 min. Based on these results, the draft scale was considered understandable and feasible for administration and was therefore applied to the main study sample. This step is consistent with scale development recommendations, which emphasize pilot testing as an essential phase for ensuring item clarity and practical applicability before conducting large-scale psychometric analyses^17^.

### Data collection method

The data were collected using the Sociodemographic Data Collection Form and T1DMS-C after explaining the study to children and parents in the endocrine outpatient clinic.

### Sociodemographic data collection form

Researchers developed the form. It comprises 14 questions to assess the sociodemographic characteristics of children with diabetes (age, gender, school attendance, year of diabetes diagnosis, family history of diabetes, and diabetes education).

### Type 1 diabetes mellitus self-management scale for children

The draft version of the T1DMS-C consisted of 46 items. Item deletion decisions were not based solely on statistical criteria. The research team also reviewed whether each item represented an essential component of diabetes self-management in children aged 7–12 years. Items that showed weak psychometric performance and overlapped conceptually with better-performing items were removed. The retained 16 items were judged to represent four clinically meaningful domains of pediatric diabetes self-management: care, treatment, nutrition, and exercise. The care subscale reflects children's general diabetes-related daily care behaviors; the treatment subscale focuses on insulin administration and treatment adherence; the nutrition subscale evaluates diet-related self-management behaviors; and the exercise subscale assesses physical activity-related self-management practices.

The scale is a five-point Likert-type scale, with a lowest score of 16 and a highest score of 80. High scores obtained from the scale indicate that children have high self-management of diabetes. The construct validity and reliability results of the scale are given in the findings section. Consistent with scale development guidelines, item reduction was performed based on psychometric evidence from factor analyses, and reliability results^16^.

### Data analysis

In the descriptive data analysis, counts, percentages, and means were used. Cronbach's alpha and McDonald's omega were used to determine the internal consistency of the scale and its subscales; Pearson correlation analysis and split-half analysis were used for the item-total score analysis of the scale and its subscales. Response bias in the scale was assessed using the Hotelling's t-test. The data were analyzed using SPSS 24.0, AMOS 24.0, and JAMOVI 2.2. Scale validity was assessed using content validity indices (I-CVI and S-CVI), exploratory factor analysis (EFA), confirmatory factor analysis (CFA), and analyses of convergent and discriminant validity. EFA was performed using principal axis factorization with a Promax rotation, applying a 1-eigenvalue criterion and a 0.20 factor-loading cutoff. Prior to CFA, the assumptions of multicollinearity and multivariate normality were examined. The variance inflation factor (VIF) and tolerance values indicated no multicollinearity (VIF<10, tolerance>0.1), and the data met the assumption of multivariate normality. Following methodological recommendations, the dataset was randomly split into two subsamples using a random allocation procedure; EFA was conducted on the first subsample and CFA on the second. This approach was taken to strengthen construct validity by reducing the risk of overfitting^16,17^. The significance level was set at 0.05.

## RESULTS

### Demographic data

A total of 50% of the children in the study were female, and 50% were male. It was found that 99.1% (n=210) of the children went to school. A total of 65.1% (n=138) went to school full-time, and 34.4% (n=73) went to school half-day. A total of 58% of the children had two or more siblings, and 87.7% (n=186) had no other chronic diseases except diabetes. A total of 43.4% of the children had no other family member with a diagnosis of diabetes; 29.2% reported that their first-degree relatives had diabetes, and 26.9% reported that their second-degree relatives had diabetes. A total of 96.2% of the children received insulin therapy, and 38.2% were hospitalized in the last year. A total of 35.8% were found to be hospitalized due to hyperglycemia. A total of 97.6% of the children reported receiving education on diabetes. Of these children, 90.1% received diabetes education from a nurse.

### Preliminary analyses

In the item reduction phase, items were evaluated using statistical and theoretical criteria, including communalities, factor loadings, cross-loadings, item-total correlations, subscale-total correlations, and conceptual relevance to pediatric diabetes self-management. After this process, the final 16-item structure was retained. To reduce the risk of overfitting, the dataset was randomly split into two subsamples; EFA was conducted in the first subsample and CFA was conducted in the second subsample.

### Exploratory factor analysis

The scale explains 57.730% of the total variance, and four factors with eigenvalues greater than 1.00 were identified. The care subscale explains 38.759% of the total variance, the treatment subscale 8.040%, the nutrition subscale 6.285%, and the exercise subscale 4.645%. The factor loadings for the care subscale ranged from 0.536 to 0.742, those for the treatment subscale ranged from 0.491 to 0. 861, the factor loadings for the nutrition subscale ranged from 0.609 to 0.863, and the factor loadings for the exercise subscale ranged from 0.380 to 0.862 (Table 1).

**Table 1:** Results of the exploratory factor analysis (n=106).

Items	Factor loads			
	Factor 1 Care	Factor 2 Treatment	Factor 3 Nutrition	Factor 4 Exercise
I administer my insulin injections exactly as I was taught. (ÇDÖYÖ 7)		0.861		
I can inject my insulin in a different place each time. (ÇDÖYÖ 8)		0.491		
I adjust my insulin dose according to my blood sugar results, as recommended to me. (ÇDÖYÖ 10)		0.697		
I know how to use the insulin pump/insulin pen prescribed for my treatment. (ÇDÖYÖ 11)		0.817		
To prevent my blood sugar from rising, I can exercise regularly, as recommended by my diabetes team. (ÇDÖYÖ 17)				0.723
I adjust my exercise routine according to my blood sugar results. (ÇDÖYÖ 18)				0.862
I stick to my diet plan. (ÇDÖYÖ 19)			0.731	
I never skip my main meals (breakfast, lunch, and dinner). (ÇDÖYÖ 20)			0.863	
Even when I eat outside the home (at restaurants, school, or visiting friends/family), I stick to my diet. (ÇDÖYÖ 22)			0.612	
I check my feet regularly (for cuts, scrapes, etc.) (ÇDÖYÖ 27)	0.742			
I keep my toenails straight and short. (ÇDÖYÖ 28)	0.716			
I wear loose-fitting and cotton socks. (ÇDÖYÖ 29)	0.779			
I prefer shoes that fit my feet properly and don't squeeze them. (ÇDÖYÖ 30)	0.616			
I go for regular check-ups with my doctor every 3 months for my diabetes. (ÇDÖYÖ 31)	0.536			
I ask my nurse or doctor about any problems I think might be related to my diabetes. (ÇDÖYÖ 34)				0.380
I can achieve the goals I've set for keeping my diabetes under control. (ÇDÖYÖ 41)			0.609	
Eigenvalue	6.579	1.723	1.405	1.157
Explained variance (%)	38.759	8.040	6.285	4.645
Explained total variance (%)	57.730			
KMO	0.840			
Bartlett χ^2^(p)	867.411 (p<0.001)			

KMO: Kaiser–Meyer–Olkin measure of sampling adequacy; ÇDÖYÖ: Type 1 Diabetes Self-Management Scale for Children original item code; χ^2^: chi-square.

### Confirmatory factor analysis

**Figure 2:**
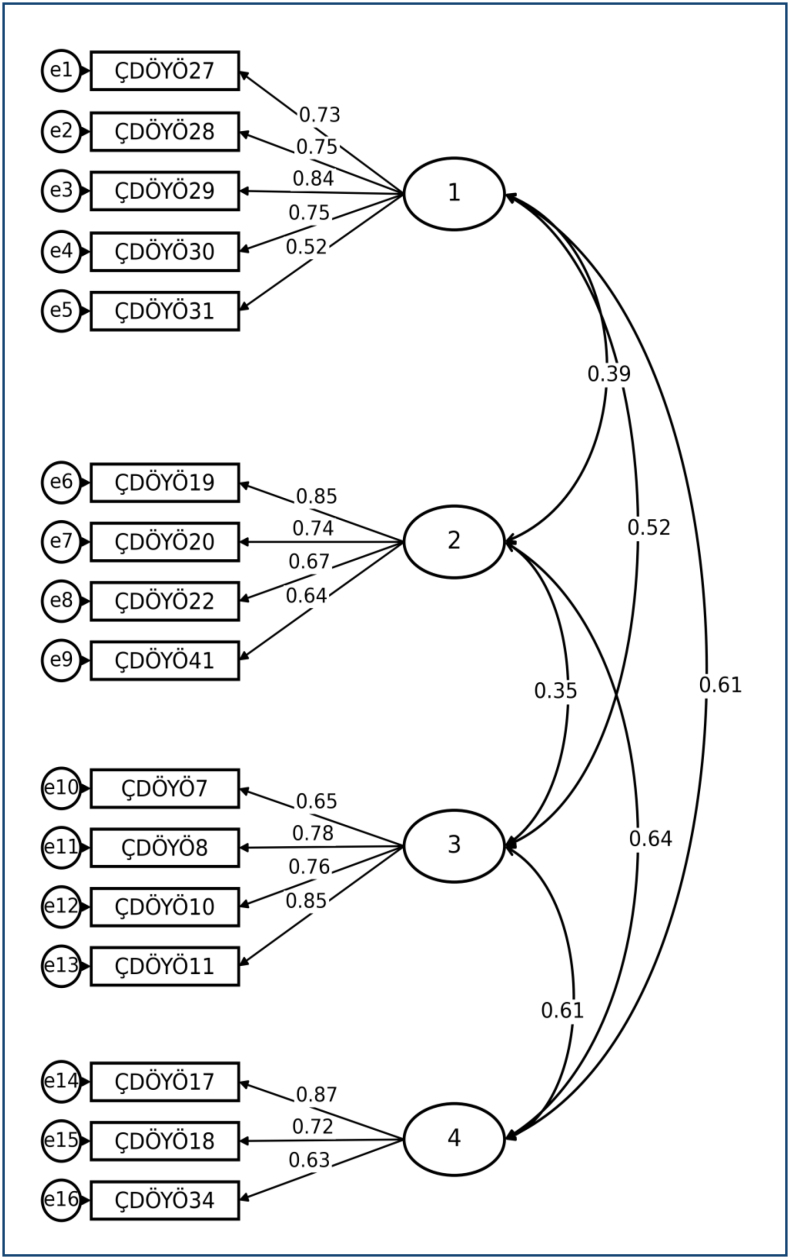
Confirmatory factor analysis diagram of type 1 diabetes self-management scale for children (n=106).

According to the CFA, the factor loadings of the care subdimension ranged from 0.52 to 0.84, those of the treatment subdimension ranged from 0.64 to 0.85, those of the nutrition subdimension ranged from 0.65 to 0.85, and those of the exercise subdimension ranged from 0.63 to 0.87 (Figure 2). For the four-factor model, the calculated chi-square value was 143.154, with 98 degrees of freedom, and the significance level was p=0.002. The chi-square/degrees of freedom ratio (χ^2^/df) was found to be 1.461. In addition, the fit indices were calculated as goodness-of-fit index (GFI)=0.901, comparative fit index (CFI)=0.939, Tucker–Lewis index (TLI)=0.926, incremental fit index (IFI)=0.941, and root mean square error of approximation (RMSEA)=0.066. These findings indicate that the model demonstrated an acceptable level of fit (Table 2).

**Table 2:** Model fit indices for confirmatory factor analysis (n=106).

	X^2^	df^a^	X^2^/df	RMSEA^b^	GFI^c^	CFI^d^	IFI^e^	TLI^f^
Four Factor Model	143.154	98	1.461	0.066	0.901	0.939	0.941	0.926

a Degrees of freedom

b Root Mean Square Error of Approximation

c Goodness of Fit Index

d Comparative Fit Index

e Incremental Fit Index

f Tucker-Lewis Index. GFI: goodness-of-fit index; CFI: comparative fit index; IFI: incremental fit index; TLI: Tucker–Lewis index; RMSEA: root mean square error of approximation

According to the CFA results, the scale's convergent and discriminant validity were examined. The analysis revealed that in all four sub-dimensions, the composite reliability (CR) value was greater than 0.70, the average variance extracted (AVE) value was above 0.50, and CR>AVE. This indicates that the scale has convergent validity. The analysis also showed that maximum shared variance (MSV)<AVE; average shared squared variance (ASV)<AVE; and the square root of AVE was greater than the interfactor correlation. These results indicate that the scale has discriminant validity (Table 3).

**Table 3:** Convergent and discriminant validity (n=106).

Subscale	CR	AVE	MSV	MaxR (H)	1	2	3	4	ASV
Care	0.846	0.529	0.375	0.868	**0.727**				0.26
Treatment	0.818	0.532	0.415	0.842	0.386**	**0.729**			0.23
Nutrition	0.846	0.581	0.369	0.861	0.516***	0.345**	**0.763**		0.25
Exercise	0.786	0.555	0.415	0.828	0.612***	0.644***	0.608***	**0.745**	0.39

CR: composite reliability; AVE: average variance extracted; MSV: maximum shared variance; MaxR(H): maximal reliability; ASV: average shared squared variance. Bold diagonal values indicate the square root of AVE for each construct.

** p < 0.01;

*** p<0.001.

### Reliability analysis

The Cronbach's alpha coefficient for the entire scale was determined to be 0.893. The Cronbach's alpha coefficients for the scale's care subscale were 0.845, for the treatment subscale 0.813, for the nutrition subscale 0.812, and for the exercise subscale 0.757. As a result of the split-half analysis, the Cronbach's alpha value for the first half was determined to be 0.817 and for the second half 0.767. The correlation between the two halves was 0.879. The Spearman-Brown coefficient was calculated as 0.936 and the Guttman split-half coefficient as 0.935. For the entire scale, the McDonald's Omega coefficient was 0.895, while the coefficients for the care subscale were 0.852, for the treatment subscale 0.820, for the nutrition subscale 0.817, and for the exercise subscale 0.773 (Table 4).

**Table 4:** Reliability analysis results (n=212).

	Cronbach's alpha	Mcdonald omega	Split half analysis					M±SD
			Part 1 Cronbach's alpha	Part 2 Cronbach's alpha	Spearman-Brown	Guttman split-half	Correlation Between Forms	
Scale total	0.893	0.895	0.817	0.767	0.936	0.935	0.879	62.11±10.11
Care subscale	0.845	0.852						20.04±4.02
Treatment subscale	0.813	0.820						16.49±3.04
Nutrition subscale	0.812	0.817						14.58±3.29
Exercise subscale	0.757	0.773						11.01±2.69

M: mean; SD: standard deviation.

A Hotelling's t-test was conducted to determine whether there was response bias in the scale, and the Hotelling's t-value was found to be 540.959, with F=58.937 and p<0.001. The analysis revealed no response bias in the scale. It was determined that the correlations between the scale items and the total scale score ranged from 0.408 to 0.660. It was determined that the correlations between the scale items and the subscale total scores ranged from 0.494 to 0.865. It was found that no single item, when removed from the scale, significantly increased Cronbach's alpha (Table 5).

**Table 5:** Item-total and item-subscale score correlations (n=212).

Items	Cronbach's alpha if item deleted	Corrected item-total correlation (r)*	Corrected item-subscale total score correlation (r)*
I1 (ÇDÖYÖ 7)	0.887	0.542	0.622
I2 (ÇDÖYÖ 8)	0.889	0.466	0.547
I3 (ÇDÖYÖ 10)	0.887	0.529	0.642
I4 (ÇDÖYÖ 11)	0.885	0.604	0.728
I5 (ÇDÖYÖ 17)	0.883	0.630	0.663
I6 (ÇDÖYÖ 18)	0.886	0.566	0.615
I7 (ÇDÖYÖ 19)	0.886	0.560	0.687
I8 (ÇDÖYÖ 20)	0.888	0.507	0.639
I9 (ÇDÖYÖ 22)	0.891	0.438	0.600
I10 (ÇDÖYÖ 27)	0.882	0.649	0.804
I11 (ÇDÖYÖ 28)	0.888	0.516	0.808
I12 (ÇDÖYÖ 29)	0.882	0.660	0.777
I13 (ÇDÖYÖ 30)	0.883	0.650	0.803
I14 (ÇDÖYÖ 31)	0.891	0.408	0.865
I15 (ÇDÖYÖ 34)	0.887	0.549	0.494
I16 (ÇDÖYÖ 41)	0.886	0.574	0.612

* Significant at p<0.001. I: item.

## DISCUSSION

Type 1 diabetes is a common chronic disease in childhood and adolescence that affects quality of life, and self-management in this process requires not only medical treatment but also the development of behavioral, psychosocial, and cognitive skills. Therefore, assessing self-management skills is important. In this study, a type 1 diabetes self-management scale for children was developed and its psychometric properties examined. The scale was found to have a four-factor structure, explaining 57.7% of the total variance. This rate indicates strong construct validity^17,19^. The results of the confirmatory factor analysis also support the good model fit^20^. Wang et al. also reached similar results in their studies^21^. One of the important contributions of our study is revealing the multidimensional structure of self-management. The four-factor structure shows that self-management is not only about adherence to treatment; it also includes cognitive, behavioral, and relational processes^22-24^.

Diabetes management in children encompasses blood glucose monitoring, insulin administration, nutrition, physical activity, and prevention of complications. The effectiveness of this process depends on age-appropriate knowledge and skills. However, psychometrically robust scales specific to children are limited in the literature. Self-management involves taking responsibility for one's illness and includes knowledge, motivation, and behaviors that improve health outcomes. Therefore, it is recommended to use both objective and subjective methods in the assessment^25^. Current models address self-management with components such as life management, insulin therapy self-management, health literacy regarding acute complications, problem-solving, self-efficacy, family support and communication, and blood glucose self-monitoring^18,26-28^. The four-dimensional structure in this study goes beyond classical compliance-based approaches, offering a more comprehensive assessment. Compared to previous scales, its multidimensional approach to self-management can contribute to more targeted planning of clinical interventions.

The findings on validity and reliability are consistent with the literature. High content validity indices indicate that the scale represents the concept well^29,30^. High Cronbach's alpha and McDonald's omega coefficients support strong internal consistency. Furthermore, split-half analysis and item-total correlations demonstrate the scale's reliability. The absence of items that, when removed, would increase reliability suggests that the items are functional^31,32^.

In recent years, there has been an increase in similar scale development studies in the literature. Küçük and Akçay Didişen developed a valid and reliable scale evaluating blood glucose measurement skills in adolescents^33^. Khalifah et al. developed a scale that evaluates transition readiness and provides multifaceted measurement^34^. These studies demonstrate the importance of age-specific tools. The role of parents is also important. Hölgyesi et al. found a relationship between parental self-efficacy and the child's glycemic control^35^. This indicates that self-management is related to family dynamics. Family-centered interventions have also been reported to be effective.

The management of acute complications such as hypoglycemia is also part of self-management. Cindoglu et al. and Benioudakis et al. have shown that scales developed in this area are reliable^36,37^. These findings support the need to address complication management as a separate component. Psychosocial dimensions are also important. The ViDa1 scale assesses quality of life and self-care^38^. Health literacy also affects self-management^39^. Studies on parental quality of life and diabetes acceptance also demonstrate the importance of psychological adjustment^40^.

In conclusion, there is a need for multidimensional, valid, and reliable scales for children with type 1 diabetes. Although current studies contribute, this need persists. The scale developed in this study appears to be a psychometrically sound and clinically useful tool for assessing diabetes self-management in children. In the future, the clinical validity can be further strengthened by examining the relationship between these dimensions and HbA1c and treatment adherence.

## CONCLUSION

This study demonstrated that the T1DMS-C is a valid and reliable self-report instrument for assessing diabetes self-management in children. The scale enables evaluation of overall self-management levels as well as specific sub-dimensions that influence self-management and may support planning targeted interventions for children with low scores. For example, a low score on the nutrition subscale may guide nurses to provide individualized carbohydrate-counting education and meal-planning counseling, whereas a low score on the exercise subscale may indicate the need for targeted counseling on safe physical activity and glucose monitoring before and after exercise. In addition, the T1DMS-C offers a standardized, psychometrically sound tool specifically developed for children aged 7–12 years, addressing an important gap in the pediatric diabetes literature, where available measures are often limited or not developmentally appropriate. By providing a multidimensional assessment of self-management behaviors, the scale may help improve clinical follow-up, identify children at risk for inadequate self-management, and support individualized education and counseling programs. Furthermore, the T1DMS-C may facilitate evidence-based nursing and multidisciplinary interventions by enabling the monitoring of behavioral outcomes over time and strengthening comparability across studies in pediatric diabetes research. Despite these contributions, the study has certain limitations. The sample was drawn from a single university hospital, which may limit generalizability. As a self-report measure, responses may have been influenced by social desirability or individual interpretation. In addition, test–retest reliability was not assessed, criterion validity could not be examined because there was no gold-standard tool for this age group, and associations with clinical outcomes, such as HbA1c, were not evaluated. Future studies should validate the T1DMS-C in larger and more diverse populations, assess test–retest reliability, and examine criterion-related validity using clinical indicators such as HbA1c, hospitalization frequency, and hypoglycemia/hyperglycemia episodes. Moreover, cross-cultural adaptation and validation studies are recommended to support international comparisons and strengthen global evidence on pediatric diabetes self-management.

### Limitations

This study has several limitations. First, the use of convenience sampling may limit the generalizability of the findings. However, this limitation may have been partially mitigated by the adequate sample size, the study being conducted in a university hospital admitting patients from different regions of the country, and the relatively homogeneous distribution of participants. Second, because the data were collected through self-reports, the findings are limited to the children's own statements. Third, the inability to examine the relationship between the scale scores and clinical/physiological indicators such as HbA1c, glycemic control, hospitalization frequency, and other diabetes-management-related outcomes limited a more comprehensive evaluation of criterion validity. In addition, the substantial reduction in the number of items during the scale development process suggests that some aspects of the construction may have remained outside the instrument's scope. Fourth, although the scale explained 57.7% of the total variance, indicating that it captures an important proportion of self-management in children with type 1 diabetes, self-management is a complex, multidimensional construct that cannot be fully explained by a single instrument. The remaining variance may be attributable to other individual, familial, environmental, and clinical factors. Therefore, although the scale appears to be a strong tool for assessing children's self-management levels, it should not be interpreted as encompassing all dimensions of the construct. Finally, because the participants were children aged 7–12 years, their responses may have been influenced by developmental level, reading comprehension, recall ability, and the desire to provide socially acceptable answers.

## ETHICAL ASPECT OF THE STUDY

This study was conducted in accordance with the principles set out in the Declaration of Helsinki. Ethics committee approval was obtained from the Pharmaceuticals and Non-Medical Device Research Ethics Committee (Decision number 2021/013). Written approval was obtained from the chief physician of the hospital where the study was conducted with the number E-14567952-900-147026. Written and verbal consent was also obtained from the children and their parents.

## Data Availability

The datasets generated and/or analyzed during the current study are available from the corresponding author upon reasonable request.
